# Mirror Visual Feedback to Improve Bradykinesia in Parkinson's Disease

**DOI:** 10.1155/2016/8764238

**Published:** 2016-08-01

**Authors:** Gaia Bonassi, Elisa Pelosin, Carla Ogliastro, Cecilia Cerulli, Giovanni Abbruzzese, Laura Avanzino

**Affiliations:** ^1^Department of Experimental Medicine, Section of Human Physiology and Centro Polifunzionale di Scienze Motorie, University of Genova, 16132 Genova, Italy; ^2^Department of Neuroscience, Rehabilitation, Ophthalmology, Genetics and Maternal Child Health, University of Genova, 16132 Genova, Italy

## Abstract

Mirror visual feedback (MVF) therapy has been applied to improve upper limb function in stroke. When combined with motor training, MVF improves the performance of the trained and untrained hand by enhancing the excitability of both primary motor cortices (M1s). Bradykinesia is a typical feature of Parkinson's disease (PD), characterized by slowness in the execution of movement. This condition is often asymmetrical and possibly supported by a volitional hypoactivation of M1. MVF therapy could tentatively treat bradykinesia since the untrained hand, which benefits from the exercise, is generally more severely impaired in undertaking sequential movements. Aim of the study was to evaluate whether MVF therapy may improve bradykinesia of the more affected hand in PD patients. Twelve PD patients and twelve healthy controls performed for 10 minutes a finger sequence, receiving MVF of the more affected/nondominant hand. Before and after MVF training, participants performed a finger sequence at their spontaneous pace with both hands. M1 excitability was assessed in the trained and untrained hemispheres by means of transcranial magnetic stimulation. Movement speed increased after MVF training in either hand of both groups. MVF therapy enhanced cortical excitability of M1s in both groups. Our preliminary data support the use of MVF therapy to improve bradykinesia in PD patients.

## 1. Introduction

Bradykinesia is one of the cardinal symptoms of Parkinson's disease (PD) and is defined as reduced speed when initiating and executing a single movement with progressive reduction of its amplitude, up to complete cessation during repetitive simple movements [1, 2]. PD motor manifestations, including bradykinesia, can begin unilaterally, typically in one limb segment, when dopamine concentrations fall below 60–70% in the contralateral striatum [3]. Throughout the course of the disease the asymmetry of major features persists in most cases; asymmetric onset has even been proposed as criteria for PD diagnosis. Generally, bradykinesia is experienced in repetitive/rhythmic voluntary movements such as finger tapping or steady gait, thus inducing motor difficulties in PD that affect almost all daily life activities [4–6]. Furthermore, PD patients may exhibit greater impairment in the speed rather than in the amplitude of movement (or vice versa), and these different phenotypes may differently respond to pharmacological treatment [7].

Beside conventional rehabilitative approaches, action observation (AO) has recently been suggested as a novel technique to improve bradykinesia in PD patients [8], with positive effects on the spontaneous rate of finger movements even after a single session of AO training [8].

Mirror visual feedback (MVF) therapy is an innovative rehabilitative approach, which attracted a growing interest in the last few years (for a review see [9]). MVF therapy aims at supplying visual feedback from the affected arm in a very peculiar way, as the subject is performing the action with the opposite nonaffected limb (thus receiving motor training and proprioception from that limb) but receives visual feedback from the affected limb [10]. MVF was originally used to alleviate phantom-limb pain after upper limb amputation [11]. Since then, the technique has been successfully applied to improve upper limb function in other neurological diseases [12, 13] and in the elderly [14]. Data from the literature suggest that MVF combined with motor skill training may improve performance of the trained and untrained hand, most likely inducing plasticity modifications in both the primary motor cortices (M1s) [15, 16].

The above-mentioned evidence paved the way for the use of the MVF therapy as a therapeutic option for treating bradykinesia in PD patients.

Indeed, one interesting feature of the MVF is that only one hand needs to be actively trained to provide performance improvement of both the trained and untrained hand [15, 17]. In PD patients with bradykinesia, the untrained hand should be on the most affected side, in which the ability to undertake sequential or simultaneous movements is severely reduced [18]. In this way training the less affected hand can improve the function of the other, more affected, hand. Further, it is worth noting that the relationship between fatigue and bradykinesia in PD patients is still under discussion. Fatigue is a common symptom in PD patients, with a reported prevalence of approximately 33% [19]. Fatigue could interfere with the outcome of a rehabilitative program [20], and therefore, assuming that a relationship between bradykinesia and fatigue exists, training the less affected side may improve the training outcome. Finally, from a neurophysiological point of view, a large body of evidence suggested that hypoactivation of M1 could be the functional correlate of PD bradykinesia [21–23].

Hence, the present study was designed (i) to investigate whether MVF therapy can influence specific aspects of bradykinesia, such as speed, (ii) to explore whether MVF was able to induce excitability changes in M1s in PD patients as already reported in healthy controls, and (iii) to elucidate whether the severity of fatigue might influence changes in motor performance induced by MVF in the trained and untrained hands.

To this end, participants underwent a “mirror training,” performing a sequential finger motor task with the less affected side for PD patients (dominant/right hand for healthy subjects (HS)) outside a mirror box. At the same time, all participants were required to carefully observe hand's movements in the mirror, in order to create the illusion of moving the more affected hand in PD patients and the left (nondominant) hand in healthy controls, thus creating visual feedback training [10]. Motor performance and cortical excitability of M1s were tested before and immediately after MVF training. Further, in patients with PD the extent of perceived fatigue was evaluated by means of Parkinson's Fatigue Scale-16.

Our hypotheses were the following: (i) MVF would induce behavioural improvements and cortical excitability changes in the untrained side in both healthy subjects and PD patients and (ii) the extent of fatigue perceived by PD patients may have a greater influence on the behavioural improvement in the trained hand compared to the untrained hand.

## 2. Materials and Methods

### 2.1. Study Design and Participants

Thirty-three participants (21 patients with Parkinson's disease (PD) and 12 healthy subjects (HS)) were recruited for this study. Twelve PD and twelve HS were involved in the main experiment (MVF training), while nine additional patients with PD were recruited for taking part in a control experiment (training without MVF). Informed consent was obtained from all participants according to our institution's policy and to the Declaration of Helsinki. The study was approved by the local ethics committee of the University of Genoa. All patients with PD (12 females and 9 males; age, 58–80 years; and mean age 72.10 ± 4.89), diagnosed according to the United Kingdom Parkinson's Disease Society Brain Bank criteria, were recruited from the outpatient Movement Disorders Clinic of the University of Genoa. All patients were in Hoehn and Yahr stages 1 to 3 and on a stable medication regimen. Disease severity was determined by means of the MDS Unified Parkinson Disease Rating Scale, Part III: Motor (MDS-UPDRS III). The following exclusion criteria were applied: (1) past history of neurological conditions other than PD, (2) deep brain stimulation, (3) Mini-Mental State Examination score < 24, (4) visual field defects, which could prevent subjects from seeing their hand reflection, and (5) severe orthopaedic problems of the upper limb. To assess bradykinesia severity we used “The Modified Bradykinesia Rating Scale” [24]. All patients suffered from more severe symptoms on one side of their body at the time of symptom onset and at the time of enrolment in this study. This side is referred to as the more affected side. In each single patient the designation of the “more affected side” was determined from the clinical history and confirmed by clinical evaluation.

Parkinson's Fatigue Scale-16 (PFS-16), a full Likert 16-items scale, was used to evaluate the extent of perceived fatigue [25]. Rating is based on feelings and experiences over the prior 2 weeks and scoring options for each item range from 1 (“strongly disagree”) to 5 (“strongly agree”). A total PFS score is calculated as the average item score across all 16 items ranging from 1 (minimum) to 5 (maximum).

A total of twelve age and gender matched healthy subjects (HS) (6 females and 6 males; age, 64–76 years; and mean age 71.5 ± 3.89) with normal neurological examination and no history of neurological disorders were recruited from the hospital staff or relatives of the patients. None of the HS had orthopaedic hand impairment or visual field defects.

All participants enrolled in this study were right-handed based on Edinburgh Handedness Inventory [26] and had no contraindication to TMS. A written informed consent was obtained from all participants. Detailed information of demographic and clinical features of all patients is shown in Table 1.

### 2.2. Experimental Paradigm

**Figure 1:**
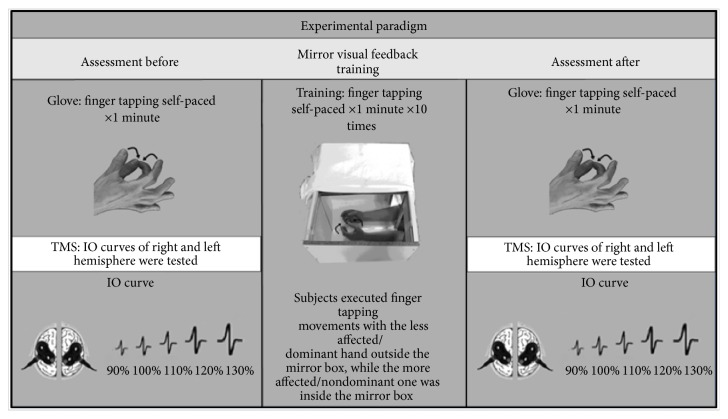
Experimental paradigm.

Motor performance and cortical excitability were evaluated before and immediately after MVF training in the main experiment and training without MVF in the control experiment. The experimental paradigm is shown in Figure 1.

### 2.3. Main Experiment: MVF Training

Motor training was performed with the use of a mirror box. A plastic collapsible triangular box with a mirror (38 cm long and 22 cm high) attached on one side was placed on the table so that the mirror would reflect one of the subject's hands while the box hid the other one from subject's view. The box had open ends to allow subjects to insert their hands [27]. Subjects were asked to hide their more affected (for PD patients)/nondominant (for HS) hand behind the mirror. The motor task consisted of ten sessions (one minute each) of finger opposition movements with the less affected/dominant hand with MVF superimposed to the other hand. To avoid fatigue, every session was alternated with one-minute rest interval.

### 2.4. Control Experiment: Training without MVF

Nine additional PD patients (control PD group) were enrolled in this control experiment. This experiment was planned to quantify the extent to which performance improvements and excitability changes in the untrained side may have occurred as a result of training of the contralateral hand by means of a mechanism of intermanual transfer. Participants were asked to place both arms inside the mirror box and to perform the same motor training using the less affected hand as in the main experimental condition. The mirror was covered with a black plastic board and participants were required to carefully watch the trained hand during training session.

### 2.5. Motor Assessment

The motor task consisted in the execution of repetitive finger opposition movements (opposition of the thumb to index, middle, ring, and little finger), for 1 minute at their spontaneous velocity with both hands, one at a time, in a random order. Motor performance was recorded by means of a sensor-engineered glove on both hands (Glove Analyzer System (GAS), ETT, S.p.A., Italy) and data were acquired at 1 kHz (National Instrument board 800008B-01).

The main outcome measure was the number of self-paced finger movements that participants were able to execute in 1 minute, whereas kinematic parameters (i.e., intertapping interval, touch duration, and percentage of correct sequences) were secondary outcome measures.

Data from glove were processed with a customized software (GAS, ETT, S.p.A., Italy) and the following parameters were computed: (i) the intertapping interval (ITI), defined as the time interval between the end of a thumb-to-finger contact and the beginning of the subsequent contact in the finger motor sequence; (ii) the touch duration (TD), the contact time between the thumb and another finger; and (iii) the Movement Rate calculated as [1/(ITI + TD)] *∗* 1000 and expressed in Hertz. The number of self-paced finger movements was calculated by multiplying the Movement Rate (expressing the number of finger touches in one second) for 60 seconds, which is the duration of the entire task. Moreover, we quantified the learning effect by measuring the increase in the number of self-paced finger movements in the assessment after MVF training with respect to the period before MVF training (Δ score of the number of fingers movements: number of finger movements/min after MVF training − number of finger movements/min before MVF training).

Finally, spatial accuracy (i.e., the ability to correctly execute the finger sequence) was investigated by calculating the percentage of correct sequences (% CORR_SEQ). The uncorrected sequences were discarded from further analysis.

In the control experiment (training without MVF), gain in motor performance of the untrained hand was quantified in the control PD group by measuring the increase in the number of self-paced finger movements in the assessment after training with respect to the period before training (Δ score of the number of fingers movements: number of finger movements/min after training − number of finger movements/min before training).

### 2.6. Cortical Excitability

Electromyographic (EMG) activity was recorded from the right and left first dorsal interosseus (FDI) muscles, with silver disc surface electrodes. The ground electrode was placed at the wrist. EMG signals were amplified and filtered (20 Hz to 1 kHz) with a D360 amplifier (Digitimer). The signals were sampled at 5000 Hz, digitized with a laboratory interface (power 1401, Cambridge Electronic Design), and stored on a personal computer for display and later offline data analysis. Each recording epoch lasted 400 ms of which 100 ms preceded the TMS stimulus. Trials with background EMG activity were excluded from analysis.

TMS was performed with a single Magstim 200 magnetic stimulator (Magstim Co., Whitland, Dyfed, UK). We determined the optimal position for activation of the left and right FDI muscles by moving the coil in 0.5 cm steps around the presumed motor hand area (referred to as “motor hot spot”). The figure-of-eight coil (wing diameters, 70 mm) was placed tangentially to the scalp with the handle pointing backward and laterally at 45° to the sagittal plane inducing a posterior anterior current in the brain. The “motor hot spot” was marked with a red wax pen by drawing a semilunar line following the anterior bifurcation of the coil and a straight line indicating the orientation of the coil handle.

At the beginning of the experiment, the stimulus intensity needed to evoke MEPs of approximately 0.8−1 mV peak-to-peak amplitude was defined (S1mV). Cortical excitability of both the left and the right M1s was tested by means of TMS Input-Output (IO) recruitment curve. During the IO curve the intensities of single TMS stimuli were expressed as a percentage of S1mV intensity. Twelve MEPs were recorded with 90%, 100% (S1mV), 110%, 120%, and 130% stimulus intensities. For each participant, the peak-to-peak MEP amplitude on single trials was used to calculate the mean MEP amplitude at each stimulus intensity. Intensities were randomly presented, in order to minimize hysteresis effects [28].

### 2.7. Statistical Analysis

We checked that variables were normally distributed (Shapiro-Wilk *W* test) and that sphericity was respected (Mauchly tests). To evaluate motor performance improvement, the mean values of the number of finger movements/min, ITI, TD, and number of correct sequences were submitted to repeated-measures ANOVA (RM-ANOVA) with time (before and after MVF training) and hand (trained and untrained) as within-subjects factors and group (PD patients and HS) as between-subjects factor. Increase in the number of finger movements/min gained after MVF training in PD patients and HS (Δ score of the number of fingers movements) was compared by means of RM-ANOVA with hand (trained and untrained) as within-subjects factor and group (PD patients and HS) as between-subjects factor. Furthermore, to investigate a possible relationship between training-induced behavioural improvement and the severity of the symptom fatigue (PFS-16 score), the correlation between Δ score of the number of fingers movements and the PFS-16 score was analyzed with Spearman's correlation coefficient. This analysis was performed for the trained and untrained hands separately.

To evaluate the effect of MVF training on IO curves, data were subjected to RM-ANOVA with time (before and after MVF training), hemisphere (trained and untrained), and TMS Intensity (90%, 100%, 110%, 120%, and 130%) as within-subjects factors and group (PD patients and HS) as between-subjects factor.

To test whether the training effect on motor performance and corticospinal excitability of the untrained side could be attributed to MVF, data from mirror and control PD groups were compared. Δ score of the number of fingers movements of the untrained hand in the mirror PD group was compared with that obtained in the control PD group by means of the unpaired Student *t*-test. IO curves data obtained from the untrained hemisphere were compared by means of RM-ANOVA with time (before and after training) and TMS Intensity (90%, 100%, 110%, 120%, and 130%) as within-subjects factors and group (mirror PD patients and control PD patients) as between-subjects factor.

*p* values of 0.05 were considered as threshold for statistical significance.* Post hoc* analysis of significant interactions was performed by means of *t*-tests applying the Bonferroni correction for multiple comparisons when necessary. Statistical analysis was performed with SPSS 22.0.

## 3. Results

### 3.1. Motor Performance

**Figure 2:**
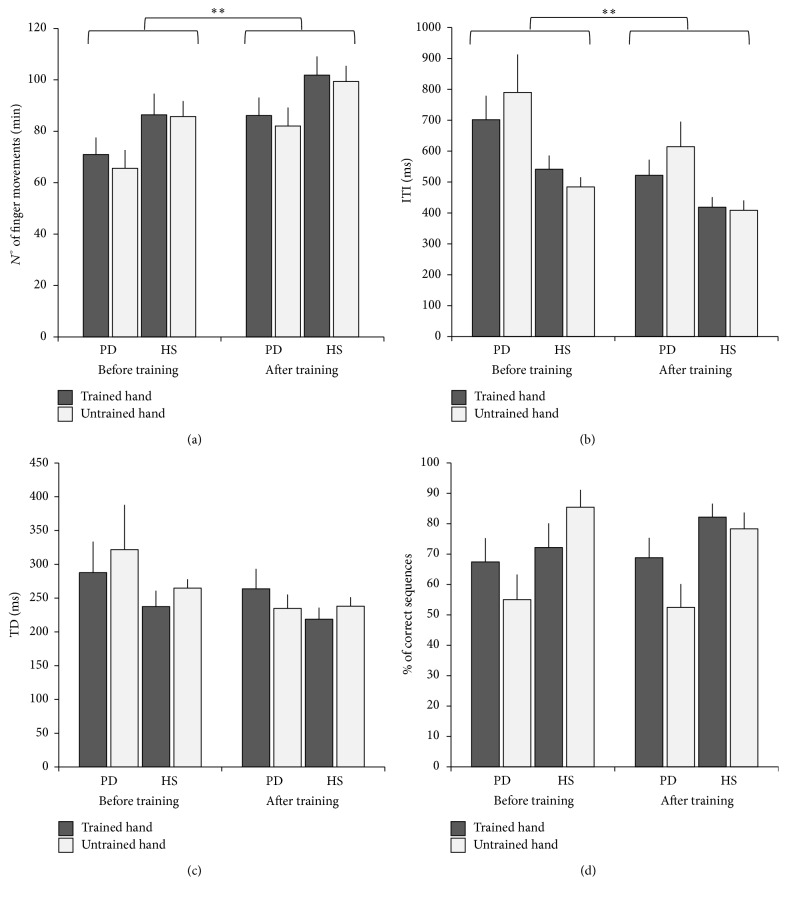
Mirror visual feedback (MVF) training effect on behavioural data. Groups (Parkinson's disease (PD) patients, healthy subjects (HS)) and hand (trained, untrained) are indicated in the abscissa. Data recorded at baseline (before training) and after MVF training session are reported. Ordinate indicates the mean values of (a) number of finger movements performed in one minute during the assessments; (b) intertapping interval expressed in milliseconds; (c) touch duration expressed in milliseconds; and (d) % of correct sequences. Vertical bars indicate standard error of the mean (SEM). Asterisks indicate that in both groups the number of finger movements performed in one minute significantly increased and ITI significantly decreased after MVF training (^*∗*^ *p* < 0.05; ^*∗∗*^ *p* < 0.01).

The number of finger movements/min increased in both the trained and untrained hand after MVF training in PD patients as well as in HS (Figure 2). Accordingly, RM-ANOVA revealed a significant effect for time (*F*_(1,22)_ = 14.36, *p* = 0.01). No significant interaction was found for time *∗* group, time *∗* hand, or time *∗* hand *∗* group (*p* always >0.05). As expected, a significant effect of group (*F*_(1,22)_ = 3.78, *p* = 0.045) was found, showing that PD patients executed a lower number of finger movements with respect to HS.

Due to the fact that no significant changes were found in touch duration (Figure 2) (RM-ANOVA: *p* always <0.05), the increased number of finger movements/min could be ascribed to the reduction of the movement time (ITI) (Figure 2). After MVF training, the mean value of ITI was significantly reduced (time, *F*_(1,22)_ = 11.98, *p* = 0.002) with no differences between groups (PD patients and HS) and between the trained and untrained hands (*post hoc* analysis time *∗* group, time *∗* hand, or time *∗* hand *∗* group, *p* always >0.05). Statistical analysis also showed that ITI was longer in PD patients with respect to HS (group, *F*_(1,22)_ = 5.89, *p* = 0.024). However, this result was mainly due to a longer value of ITI observed in the untrained (more affected) hand of PD patients with respect to HS. Indeed, RM-ANOVA showed a significant hand *∗* group interaction (*F*_(1,22)_ = 4.96, *p* = 0.036) and* post hoc* comparison revealed that ITI was significantly longer in the untrained hand in PD patients than in HS (*p* = 0.018).

At the end of the experimental protocol, the number of correct sequences increased only in the trained hand (Figure 2).* Post hoc* comparison on time *∗* hand interaction (*F*_(1,22)_ = 7.22, *p* = 0.01) showed a significant increase of the number of correct sequences in the trained with respect to the untrained hand (*p* = 0.019) after MVF training. Overall, the number of correct sequences was lower in PD participants than in HS (RM-ANOVA: group, *F*_(1,22)_ = 5.81, *p* = 0.025) with a significant hand *∗* group interaction (*F*_(1,22)_ = 4.56, *p* = 0.04).* Post hoc* analysis showed that the number of correct sequences was lower in PD subjects with respect to HS in the untrained (more affected) hand (*p* = 0.006), but not in the trained one (PD subjects versus HS, *p* = 0.29).

**Figure 3:**
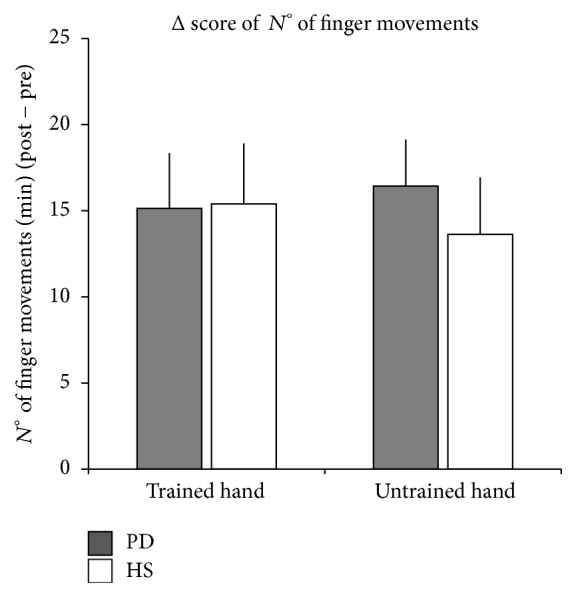
Behavioural data, showing Δ score of the numbers of finger movements performed in one minute (number of finger movements/min after MVF training − number of finger movements/min before MVF training) in the trained and untrained hands of both Parkinson's disease (PD) patients (grey bars) and healthy subjects (HS) groups (white bars). Vertical bars indicate standard error of the mean (SEM).

**Figure 4:**
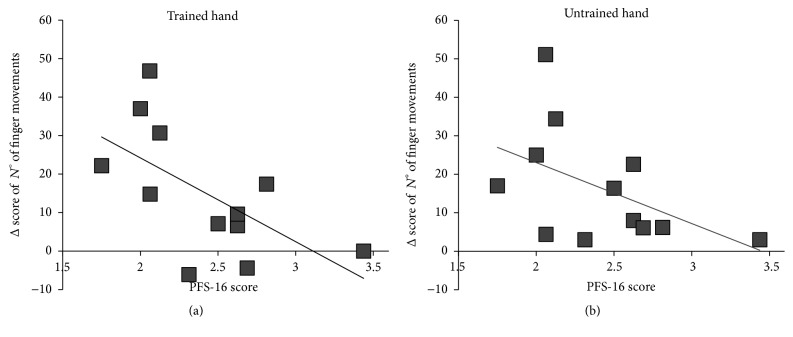
Correlation analysis between individual changes in the number of finger tapping movements induced by mirror visual feedback practice and individual scores at the Parkinson's Fatigue Scale-16 (PFS-16) questionnaire in Parkinson's disease patients. There is a significant positive correlation between the improvement in the less affected/trained hand (a) and the clinical score (*r* = 0.64; *p* = 0.024), indicating that the higher the fatigability, the lower the performance improvement. In (b) the nonsignificant correlation between the improvement of the more affected/untrained hand and PFS-16 is depicted (*r* = 0.54, *p* = 0.07).

Finally, when comparing Δ score of the number of fingers movements, RM-ANOVA showed no significant effect of hand, group, or hand *∗* group interaction (*p* always >0.05), indicating that performance gain was similar in both hands for both groups (Figure 3). However, when this Δ score was correlated with PFS-16 clinical score, a significant correlation was found only for the trained hand (trained hand: Spearman rho = 0.64, *p* = 0.024; untrained hand: Spearman rho = 0.54, *p* = 0.07), indicating that the higher the fatigue symptom the lower the performance improvement (Figure 4).

### 3.2. Cortical Excitability

**Figure 5:**
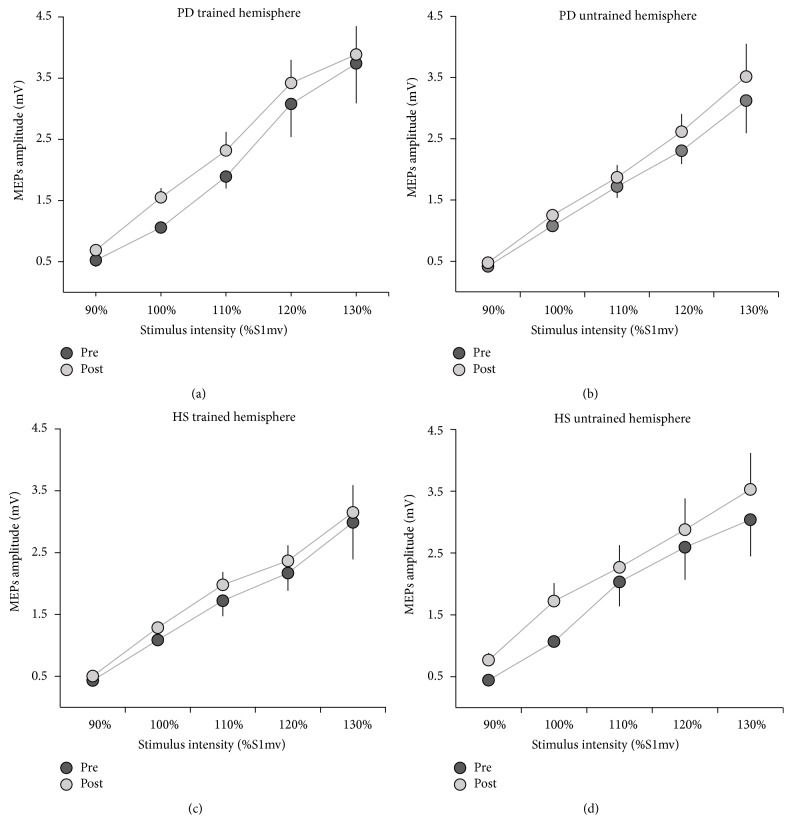
Input-Output (IO) curves measured in the first dorsal interosseus (FDI) muscle, of the trained (a and c) and the untrained (b and d) M1s before and after mirror visual feedback (MVF) training. Data of both groups, Parkinson's disease (PD) patients (a and b) and healthy subjects (HS) (c and d), who underwent MVF training, are shown. MEP amplitudes, in mV, are depicted from 90% to 130% S1mV (the stimulus intensity needed to evoke MEPs of approximately 0.8−1 mV peak-to-peak amplitude). Vertical bars indicate standard error of the mean (SEM).

For the IO curve the RM-ANOVA showed a significant effect of time (*F*_(1,22)_ = 14.57, *p* < 0.01) and intensity (*F*_(4,88)_ = 63.51, *p* < 0.01) (Figure 5). The excitability of each hemisphere, as tested with the IO curve, significantly increased in PD patients and in HS, with no difference between groups (*F*_(1,22)_ = 0.26, *p* = 0.61) after the MVF training. The comparison between the trained and untrained hemispheres did not show any significant difference (*F*_(1,22)_ = 0.24, *p* = 0.62), demonstrating that cortical excitability in both hemispheres was similarly modified.

### 3.3. Control Experiment

**Figure 6:**
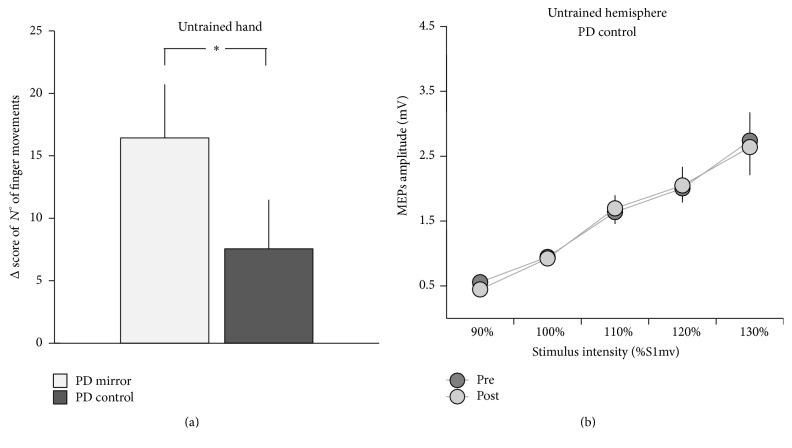
Data from the control experiment (training without MVF). (a) shows the behavioural data, expressed as performance gain (Δ score of the numbers of finger movements performed in one minute) in the untrained hand of PD patients enrolled in the main experiment (PD mirror) and PD patients enrolled in the control experiment (PD control). In (b) the Input-Output (IO) curves of the untrained M1 of the PD control group, before and after training, are depicted. MEP amplitudes, in mV, are depicted from 90% to 130% S1mV (the stimulus intensity needed to evoke MEPs of approximately 0.8−1 mV peak-to-peak amplitude). Vertical bars indicate standard error of the mean (SEM). ^*∗*^ *p* < 0.05.

Motor performance was associated with a larger gain (higher value of Δ score of the number of fingers movements) in the untrained hand in the mirror PD group with respect to the control PD group (*p* = 0.041) (Figure 6(a)). Further, when comparing the IO curves of the untrained hemisphere, RM-ANOVA showed a significant group *∗* time interaction (*F*_(1,19)_ = 4.17; *p* = 0.035), indicating that the excitability of the untrained hemisphere significantly increased after training only in the mirror PD group (*p* = 0.012) and not in the control PD group (*p* = 0.77) (Figure 6(b)).

## 4. Discussion

The main aim of the present study was to investigate whether unilateral hand training performed by PD patients with the less affected side and based on MVF was able to induce changes in bradykinesia of the untrained (and more affected) hand. Further, we wanted to investigate whether changes in motor performance were accompanied by changes in the excitability of the trained and untrained M1s. Finally, we wanted to disclose whether the use of MVF might reduce the impact of fatigue on the training-induced improvement of the more affected side in PD patients.

Our main findings were the following: (1) training based on MVF induced an increased number of finger movements, performed in one minute, of the untrained hand in PD patients similarly to HS; (2) this behavioural improvement was associated with the facilitation of excitatory function of the corticospinal pathway, which increased the MEP amplitude in PD patients similarly to HS; and (3) the extent of fatigue perceived by PD patients had a greater influence (negative correlation) on the behavioural improvement in the trained hand compared to the untrained one.

The interesting feature of MVF is that only one hand needs to be actively trained to provide performance improvements of both hands [15, 17]. Here we took advantage of this feature to establish a proof of evidence on the use of MVF therapy to improve finger movements' bradykinesia in PD patients. Our PD patients performed a lower number of finger movements in one minute compared to healthy subjects and this behaviour was related to a longer time spent on movement execution (documented by greater intertapping interval values). In contrast, the time for the finger touching phase (touch duration), which is the combination of the time used for sensory processing and motor preparation, was not different between PD patients and HS.

After training, all participants increased movement speed by reducing the intertapping interval in both the trained and untrained hand and not by changing the touch duration. This finding might suggest that MVF training was able to provide information mainly dealing with the dynamic part of the movement (transition from a finger to the following one in the sequence). This emerging result is in accordance with a previous finding of our group, showing that also when trained with a video showing finger opposition movements (action observation training), PD patients improved bradykinesia by reducing the duration of the time devoted to movement execution [8]. Indeed, a possible mechanism of action of MVF involves the mirror neuron system. In addition to the different cerebral areas involved in the mirror neuron system, the superior temporal gyrus was activated during observation of actions done by others (for a review, see [29–31]) as well as during MVF intervention [32], suggesting a link between MVF and action observation.

Another finding that deserves to be discussed is that in both PD and HS groups the number of correct sequences increased after MVF training in the trained hand but not in the untrained one. Thus, if our findings support the use of MVF therapy to improve slowness of movement execution in PD patients, MVF does not seem useful for improving spatial accuracy of the untrained hand. One possible explanation may deal with the fact that physical practice and MVF training activate different sensorimotor mechanisms. Physical practice involves both motor and sensory processes [33], as the somatic sensory feedback plays a pivotal role in movement refining [34]. On the other hand, MVF does not supply somatic sensory feedback, but it is based exclusively on a visual feedback. We can suppose that visual information obtained through MVF was able to induce changes only in the dynamic part of movement, similarly to what is described for action observation [8].

The last behavioural finding of our study concerns the negative impact of fatigue on bradykinesia improvement that was more evident in the trained hand with respect to the untrained one. Fatigue is one of the most disabling nonmotor symptoms for people with PD and it has been demonstrated to severely impact quality of life. In this study, we found that the greater the severity of subjective fatigue was (according to PFS-16 score), the less the improvement in bradykinesia of the trained hand was. This finding fits well with evidence in the literature that PD patients show increased physical fatigue during a finger tapping task and a force decline during a maximum voluntary contraction [35]. Furthermore, although data in the literature are controversial, a relationship between fatigue and the sequence effect [18, 36], which represents one of the main features of bradykinesia, has been hypothesized. Our preliminary data may suggest that fatigue can influence the outcome of a training protocol, based on the repetition of sequential movements, aimed at improving bradykinesia. Since no significant correlation was found between fatigue and bradykinesia improvement in the untrained hand, we may suggest the use of MVF as rehabilitative approach in PD patients with fatigue.

It is worth noting that, in addition to behavioural results, the present study showed that M1 cortical excitability was significantly enhanced after MVF training. The Input-Output recruitment curve refers to a global measure of excitability of the corticospinal pathway [37], reflecting not only the number of firing neurons activated by the suprathreshold stimuli but also the neuronal excitability produced by the subthreshold stimuli [38]. Robust evidence in the literature obtained in healthy controls already showed that a possible mechanism of action of MVF deals with increased excitability not only of the trained M1 but also of the untrained one [14, 15]. It has already been hypothesized that the increased M1 excitability in the untrained hemisphere might have been caused by the M1 mirror neuron system-like properties or via dorsolateral prefrontal cortex activation [9, 15]. Indeed, MVF creates an intermodal conflict between visual and proprioceptive and tactile senses. The right dorsolateral prefrontal cortex was primarily activated by discrepancies between signals from sensory systems [39]. However, regardless of either mechanism, we found that in PD patients, similarly to HS, the effect of MVF is probably related to the induction of cortical excitability changes in M1.

Finally, we can reasonably think that performance and excitability changes in the untrained side occurred as a result of MVF. Data from the control experiment in PD patients showed that behavioural changes were greater when training was associated with MVF with respect to motor training alone, while the excitability of the untrained hemisphere significantly increased only after MVF training. Indeed, it is worth noting that learning how to perform a motor task with one hand can result in performance improvements in the other hand, a process called intermanual transfer [40–42]. Our results are in line with a recent study [43] showing that although motor performance significantly increased in the untrained hand in both conditions (with and without MVF), the overall improvement was greater in the mirror group with respect to the control group. Our hypothesis is that MVF-associated improvement may derive from the combination of performance gain induced by intermanual transfer (likely via interhemispheric mechanisms)* plus* performance gain induced by visual feedback (likely via action observation mechanisms).

In accordance with this hypothesis we showed that training based on MVF may influence the excitability of the transcallosal pathway similarly to training without MVF [16]. Further, we can suppose that action observation mechanisms are involved only during training with MVF since observational learning has been demonstrated to be highly effector-dependent [44]. It has been shown that finger sequence learning based on observation of right hand performance did not transfer to the left hand [44].

There are some study limitations that should be acknowledged. First, the observed positive effect of MVF training was obtained in a relatively small sample that is not necessarily representative of the whole PD population. Second, in this pilot study, we tested the effect of a single training session on improving finger movements; a longer period of training should be examined. Third, our experimental protocol was designed to study immediate changes of MVF training and we did not assess long-lasting effects.

## 5. Conclusions

In this proof-of-concept study, we have provided novel evidence that MVF training might induce improvement in finger movements' bradykinesia of the more affected (untrained) hand in PD patients. We have also shown that the final common pathway for the effect of MVF was the change of excitability in M1. Further, our findings support the idea that fatigue could impact behavioural improvement in the trained hand more than in the untrained one even if our data have been obtained in a relatively small sample. However, if true, this finding may be relevant for future clinical studies that aim to improve bradykinesia in PD patients suffering from fatigue.

## Figures and Tables

**Table 1:** Demographics and clinical characteristics of PD patients.

Group	Patient	Age	Gender	H&Y	MDS-UPDRS III	MBRS	MBRS	PFS-16
		(years)			(score)	(untrained hand)	(trained hand)	
M	1	58	F	1.5	13	3	0	2.63
M	2	80	M	3	34	8	6	3.63
M	3	68	F	3	31	5	2	2.69
M	4	73	M	2	34	2	1	2.31
M	5	69	M	3	51	8	6	2.13
M	6	75	F	3	20	3	1	2.81
M	7	71	F	2.5	23	4	2	2.06
M	8	72	F	2	12	6	4	2.63
M	9	74	M	2.5	21	4	2	1.75
M	10	75	M	3	27	4	3	2.06
M	11	66	F	2	16	4	3	2.50
M	12	74	F	2.5	35	7	5	3.44

	Mean	71.25		2.5	26.42	4.83	2.92	2.55

C	1	68	M	2	23	3	2	2.25
C	2	72	F	2	15	4	2	1.25
C	3	80	F	2.5	37	8	2	1.75
C	4	70	M	2	29	7	5	1.81
C	5	72	F	2	12	6	4	2.63
C	6	74	F	2.5	35	7	5	3.44
C	7	78	F	2	28	6	3	2.06
C	8	71	M	2	23	7	5	1.63
C	9	74	M	2.5	27	5	3	2.19

	Mean	73.22		2.16	25.44	5.88	3.44	2.11

PD, Parkinson's disease; MDS-UPDRS III, MDS Unified Parkinson Disease Rating Scale, Part III: Motor; H&Y, Hoehn and Yahr stage; MBRS, Modified Bradykinesia Rating Scale; and PFS-16, Parkinson's Fatigue Scale-16.
